# Contraceptive use intention among women in Pakistan: Application of theory of planned behavior

**DOI:** 10.1371/journal.pone.0344246

**Published:** 2026-03-05

**Authors:** Zoya Waqas, Aisha Irum, Muhammad Ibrahim, Maheen Sughra, Sanaa Khan, Ayesha Khan, Adnan Ahmad Khan

**Affiliations:** 1 Research and Development Solutions, Islamabad, Pakistan,; 2 Akhter Hameed Khan Foundation, Islamabad, Pakistan; University of Central Punjab, PAKISTAN

## Abstract

**Introduction:**

Unintended pregnancies remain a major public health concern globally and in Pakistan, where family planning (FP) uptake continues to be hindered by entrenched social and behavioral barriers. This study applies the Theory of Planned Behavior (TPB) to examine how attitudes, subjective norms, and perceived behavioral control (PBC) shape women’s contraceptive intentions in Pakistan.

**Methods:**

We analyzed data from 13,335 non-pregnant women aged 15–49 using the Pakistan Demographic and Health Survey (PDHS) 2017–18. Partial Least Squares Structural Equation Modeling (PLS-SEM) was used to test TPB pathways. Model reliability, validity, and fit were assessed using Composite Reliability, Average Variance Extracted, discriminant validity indices, and bootstrapped estimates to ensure analytical rigor.

**Results:**

Over half of respondents lacked formal education, and most (84%) were unemployed. Only 23% had FP knowledge, and 96% were unaware of contraceptive side effects. Subjective norms negatively influenced contraceptive intentions (β = −0.056, p < 0.001), while perceived behavioral control had a positive effect (β = 0.091, p < 0.001). Attitudes showed no significant effect. These findings indicate that women’s reproductive choices are shaped more by social expectations and decision-making autonomy than by personal evaluations of contraception.

**Conclusion:**

The study demonstrates the applicability of TPB for understanding contraceptive intentions in a collectivist, patriarchal context. Subjective norms and PBC emerge as critical determinants, underscoring the need for interventions that engage families, strengthen women’s autonomy, and improve access to FP services. The findings offer a theoretically grounded and policy-relevant framework for designing behaviorally informed family planning programs in Pakistan.

## 1. Introduction

Unintended pregnancies remain a major global public health concern, with approximately 40% of the 210 million pregnancies each year classified as unintended. Nearly half of these end in abortion, 13% in miscarriage and 38% are carried to term [1]. Globally, 11% of all births occur among adolescents aged 15–19, many of whom face heightened risks of obstetric complications. In Pakistan, a low- and middle-income country (LMIC), 5% of births are unwanted, and 7% mistimed, while more than 1 in 10 pregnancies (13%) result in miscarriages, 2% in stillbirth and 2% in abortions [2]. These outcomes contribute to low birth weight, underutilization of reproductive healthcare services, infertility, and elevated maternal mortality [3,4].

Family planning is a central strategy to prevent unintended pregnancies and improve maternal and child health. Despite longstanding investments, Pakistan’s contraceptive prevalence rate remains low at 34%, and 17% of currently married women report an unmet need for family planning [5,6]. These persistent gaps suggest that barriers are rooted not only in healthcare supply constraints but also in deeply embedded social, cultural, and behavioral factors that shape women’s reproductive decisions [7,8].

**Fig 1:**
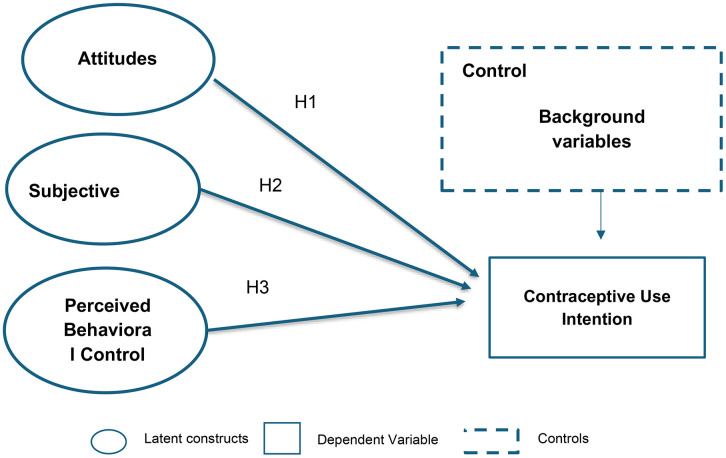
Conceptual framework. Fig shows relationships between latent, control and dependent variables.

To understand these behavioral determinants, researchers increasingly draw upon social-psychological theories [9]. The Theory of Planned Behavior (TPB) is widely used to explain health-related behaviors by positing that intentions are shaped by three psychosocial constructs: attitudes toward the behavior, subjective norms, and perceived behavioral control (Fig 1) [10]. Attitudes capture an individual’s positive or negative evaluation of performing a behavior, subjective norms reflect perceived social pressures from family, peers, or community; and perceived behavioral control refers to confidence in one’s ability to perform the behavior, accounting for internal and external constraints.

A growing body of evidence from LMICs demonstrates that TPB constructs predict contraceptive intension and use. Studies in Ghana, Indonesia, and Nigeria show that favorable attitudes, supportive social norms, and higher self-efficacy increase contraceptive uptake, while restrictive norms and low perceived control limit use [11,12]. In Pakistan, existing research highlights limited decision-making autonomy, spousal influence, misinformation, son preference as major barriers to family [7,13]. However, these studies have not systematically operationalized TPB constructs nor tested them within a comprehensive theoretical framework.

Applying TPB in the Pakistan raises important theoretical and practical questions Although TPB assumes that individuals form intentions based on personal beliefs, decision-making in Pakistan is strongly embedded within collectivist family structures, patriarchal norms, and hierarchical authority. These sociocultural dynamics may override individual attitudes, amplifying the influence of subjective norms and diminishing the role of perceived control. This creates tension between TPB’s theoretical expectations and the sociocultural realities of reproductive decision-making. Examining TPB within this setting therefore allows for a critical test of whether the model’s psychosocial pathways hold, weaken, or require contextual reinterpretation.

This study addresses these gaps by applying a TPB-based measurement and structural framework to nationally representative data from the Pakistan Demographic and Health Survey (PDHS 2017–18). The contribution of this analysis lies in (1) systematically operationalizing TPB constructs using DHS indicators, (2) empirically testing their predictive validity in a context with strong sociocultural constraints, and (3) generating insights to guide behaviorally informed, culturally grounded FP interventions.

Guided by the Theory of Planned Behavior, this study addresses the following research questions:

1. How do women’s attitudes toward contraception influence their intention to use it?
2. How do subjective norms and perceived social expectations shape contraceptive intentions?
3. How does perceived behavioral control influence women’s intentions to use contraception?
4. To what extent do these psychosocial factors collectively predict contraceptive intention in Pakistan?

By linking theory to practice, this study aims to deepen understanding of the behavioral drivers of contraceptive decision-making and inform FP programming tailored to the sociocultural realities faced by Pakistani women.

## 2. Methodology

### 2.1. Study design and research paradigm

This study adopts a quantitative, theory-driven research design grounded in the positivist paradigm, which emphasizes empirical testing of theoretically informed hypotheses. Guided by the Theory of Planned Behavior (TPB), the analysis seeks to quantify the relationships between psychosocial constructs—attitudes, subjective norms, and perceived behavioral control—and women’s contraceptive intentions in Pakistan. A positivist orientation is appropriate because TPB specifies directional causal pathways that can be empirically examined using structural models. The aim is not only to describe patterns but to assess whether TPB’s theoretical mechanisms are supported within Pakistan’s sociocultural context.

### 2.2. Study sample and data source

Data were drawn from the Pakistan Demographic and Health Survey (PDHS 2017–2018), conducted by the National Institute of Population Studies (NIPS). This study uses publicly available secondary data from the Pakistan Demographic and Health Survey (PDHS) 2017–18. Although the PDHS 2017–18 is not newly collected, it remains the most recent nationally representative survey available. The contribution of this study lies not in primary data collection but in the novel application of the Theory of Planned Behavior as a validated measurement and structural framework to examine contraceptive intentions at a national scale.

The PDHS employs a stratified two-stage sampling design using the 2017 Pakistan Population and Housing Census as the sampling frame. In the first stage, 580 enumeration blocks (EBs) were selected using probability proportional to size (PPS) across 16 urban-rural strata. In the second stage, 28 households per EB were selected via systematic random sampling, yielding a nationally representative sample of approximately 16,240 households. For this study, we included 13,335 non-pregnant women aged 15–49 years was selected. Non-pregnant women were selected to ensure that the outcome variable—intention to use contraception—was meaningful and relevant to respondents’ current reproductive decision-making.

### 2.3. Ethical considerations

The PDHS dataset is publicly available and anonymized. Permission for data use was obtained from the DHS Program. Because this study used secondary data, no additional ethical approval was required.

### 2.4. Variables

#### 2.4.1. Outcome variable.

The primary outcome variable was women’s intention to use contraception, measured by the survey question: “Do you intend to use contraceptives in the future?” Responses were coded as binary: ‘Yes’ = 1, ‘No’ = 0.

#### 2.4.2. Latent TPB constructs.

The main independent variables were the three latent constructs of the Theory of Planned Behavior: **attitudes**, **subjective norms**, and **perceived behavioral control** (PBC). All latent constructs were measured using binary indicators (0 = absence, 1 = presence) derived from survey responses.

- **Attitudes:** Assessed women’s beliefs regarding the feasibility and acceptability of contraceptive use, including perceptions of inconvenience and general acceptance or opposition to family planning. Positive attitudes were coded as 1 if women reported favorable beliefs about contraceptive use, and 0 otherwise.
- **Subjective Norms:** Captured the influence of social factors, such as partner opinions and perceived religious teachings, on contraceptive decision-making. Women who perceived supportive social expectations for contraceptive use were coded as 1; those who perceived opposition or discouragement were coded as 0.
- **Perceived Behavioral Control (PBC):** Measured respondents’ autonomy in making contraceptive decisions and their access to reproductive health services, including visits from health workers or availability of family planning services. Women were coded as 1 (autonomous) if they made contraceptive decisions either independently or jointly with their husband, and 0 if decisions were made solely by their husband or others.

Although DHS data do not provide multi-item psychometric scales, prior studies have demonstrated the feasibility of constructing binary reflective indicators from DHS items for SEM-based TPB analysis in LMICs. This approach allows meaningful quantification of psychosocial determinants using the best available population data.

#### 2.3.3. Control variables.

To account for potential confounders, the model included demographic and socioeconomic factors: employment status, occupation, wealth, education, family planning knowledge, fertility preferences, and pregnancy history. These variables provide a comprehensive understanding of factors influencing contraceptive intentions in Pakistan.

### 2.4. Analytical strategy

All statistical analyses followed established Partial Least Square Structural Equation Model (PLS-SEM) procedures, including reliability and validity assessments, multicollinearity checks, model fit evaluation, and bootstrapping with 5,000 resamples to generate robust estimates. The large sample size (13,335 women) ensures sufficient statistical power and stability of the model. PLS-SEM is suitable for:

- models with complex relationships among latent constructs;
- datasets with binary/reflective indicators;
- theory-testing in large samples;
- prediction-oriented research objectives.

This makes PLS-SEM appropriate for evaluating psychosocial pathways in population-level reproductive health data [14]. The analysis proceeded in two stages:

#### 2.4.1. Measurement model.

The measurement model (or the outer model) assessed reliability and validity of the latent constructs. It involves Confirmatory Factor Analysis (CFA) to assess the reliability and validity of the observed indicators in measuring the latent constructs. The reliability and validity of the measurement model are meant to ensure sufficient differentiation between the constructs. Internal consistency was evaluated using Composite Reliability, with thresholds of 0.7 or higher [15]. Both Subjective Norms (0.93) and Perceived Behavioral Control (0.69) met this criterion, indicating good internal consistency. Construct validity was assessed using Average Variance Extracted (AVE), with a recommended cutoff value of 0.5 or greater [16]. Attitudes (0.49), subjective norms (0.51), and perceived behavioral control (0.54) met this threshold, indicating favorable validity as highlighted in Table 1.

**Table 1:** Composite reliability and Average Variance Extracted (AVE).

Construct	Composite Reliability	Average Variance Extracted (AVE)
**Attitudes**	0.15	0.49
**Subjective Norms**	0.93	0.51
**Perceived Behavioral Control**	0.69	0.54

Furthermore, discriminant validity evaluates whether the degree to which the items used to measure different constructs are distinct from each other. Sufficient discriminant validity is achieved when the square root of the average variance extracted (AVE) exceeds the correlation coefficients between constructs. Table 2 shows the correlation matrix, with diagonal values representing the square root of the AVE and off-diagonal values indicating correlation coefficients. Henseler et al (2015) introduced the Heterotrait-Monotrait (HTMT) ratio for assessing discriminant validity, with values below 0.9 indicating adequacy. In this study, the highest HTMT value was 0.259, demonstrating favorable discriminant validity for the proposed model.

**Table 2:** Analysis of discriminant validity (Heterotrait – Monotrait ratio).

Contraceptive Behavior	Attitudes	Subjective Norms	Perceived Behavioral Control
**Attitude**	0.243		
**Subjective Norms**	0.090	0.295	
**Perceived Behavioral Control**	0.375	0.507	0.263

Furthermore, the model’s goodness of fit was evaluated using Standardized Root Mean Square Residual (SRMR), which is defined as the square root of the sum of squared differences between a model and an empirical correlation matrix, with values below 0.1 indicating a good fit [17]. The SRMR for this study’s model is 0.05, suggesting an acceptable fit.

Additionally, the Normed Fit Index (NFI) was also employed to assess model fit. The NFI evaluates the extent to which the hypothesized model fits the observed covariance structure compared to a baseline model, with values closer to 1 indicating a better fit [15,18]. The NFI for this study’s model is 0.83, highlighting a good fit. Overall, these indicators, summarized in Table 3, confirm that the model has an acceptable fit.

**Table 3:** Analysis of model fit for the TPB model.

Statistic	Value-Obtained
SRMR	0.05
NFI	0.83

#### 2.4.2. Structural Model.

The structural model, also referred to as the inner model, evaluates the hypothesized paths from attitudes, subjective norms, and perceived behavioral control to contraceptive intention. **Bootstrapping (5,000 resamples)** was used to obtain robust standard errors and significance levels. **Path coefficients (β)** quantify the strength and direction of associations. **Variance Inflation Factor (VIF)** values were examined to ensure no multicollinearity among predictors (threshold < 5). The outcomes of the structural model analysis are presented in *Fig 3* and Table 5.

## 3. Results

### 3.1. Descriptive statistics

Among the sample of women, 19.5% were aged 25–29 years, and 21.4% were from low socioeconomic backgrounds. Half (50.6%) of them had no formal education, and 84% were not currently working, with 83.7% never having worked after marriage. Most women (76.8%) lacked knowledge about family planning, and 95.9% were unaware of contraceptive side effects. 53.7% had given birth in the last five years (Table 4).

**Table 4:** Background characteristics of female respondents.

Characteristics	N	*n* (%)
Age of Women (Years)		
15-19	549	4.1
20-24	1,761	13.2
25-29	2,593	19.5
30-34	2,548	19.1
35-39	2,547	19.1
40-44	1,784	13.4
45-49	1,533	11.7
Women’s Education		
No Education	6,743	50.6
Primary Education	1,899	14.2
Secondary Education	2,740	20.7
Higher Education	1,953	14.7
Employed after marriage		
Employed	2,169	16.3
Unemployed	11,160	83.7
Employment Status		
Professionally employed	471	3.5
Self-employed	475	3.6
Clerical	9	0.1
Sales	64	0.5
Skilled manual	720	5.4
Unskilled manual	111	0.8
Un-employed	11,196	84.0
Wealth Status		
Poorer	2,854	21.4
Poorest	2,488	18.7
Middle	2,630	19.7
Richer	2,544	19.2
Richest	2,809	21.1
Number of Births in last five years		
No births	6,176	46.3
1	3,899	29.2
2	2,631	19.7
3	578	4.3
4	49	0.4
5	2	0.0
Knowledge about family planning		
Yes	3,092	23.2
No	10,243	76.8
Knowledge about side effects		
Yes	547	4.1
No	12, 788	95.9

**Table 5:** PLS results of the research model.

Variables	Path coefficients	P value
**Attitudes**	−0.175	0.294
**Subjective Norms**	−0.056*	<0.001
**Perceived Behavioral Control**	0.091*	<0.001
**Age**	−0.059*	<0.001
**Socio-economic Status**		
Poorer	0.010	0.311
Middle	0.0019	0.356
Richest	−0.021	0.058
**Education**	0.040*	<0.001
**Employment after marriage**	0.0019	0.497
**Employment**		
Professionally employed	0.010	0.582
Self-employed	0.035	0.067
Unemployed	0.027	0.064
**Number of births in the last five years**	0.032*	<0.001
**Ideal number of sons**	−0.025*	<0.001
**Total no. of children**	0.027*	<0.001
**Knowledge of Family Planning**	0.049*	<0.001
**Knowledge of Side effects**	−0.069*	<0.001

**Fig 2:**
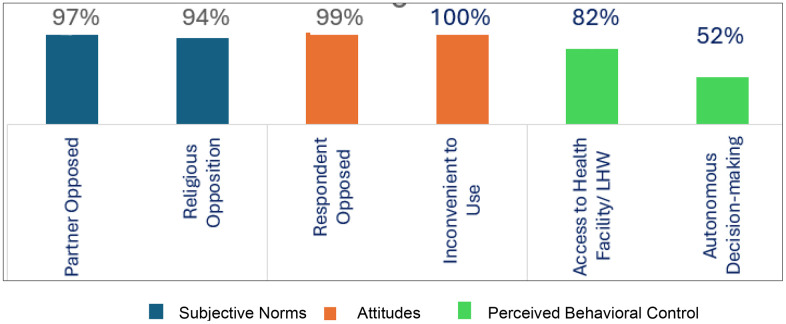
Latent constructs and the frequency of their manifest variables.

Fig 2 below illustrates the latent constructs and the frequency of their manifest variables. Subjective norms were evaluated through the influence of partners and religious beliefs on contraceptive use decision-making. The results indicate that only 2.9% of husbands opposed the use of contraceptives. Additionally, 5.85% of respondents viewed religion as a barrier, underscoring the significant role of societal values in shaping subjective norms around family planning services. In this study, attitudes were reflected in personal beliefs regarding contraceptive use and its perceived feasibility. Consequently, only 0.55% of women opposed contraceptive use, while 0.38% considered them ineffective. Furthermore, access to health facilities or Lady Health Workers (LHWs) and the participant’s degree of autonomy in contraceptive decision-making were used to measure perceived behavioral control. The findings revealed that 81.8% of respondents had access to health facilities, and 52% had autonomy over reproductive decision-making, indicating a high level of perceived behavioral control concerning the utilization of family planning services.

### 3.3. Empirical analysis

**Fig 3:**
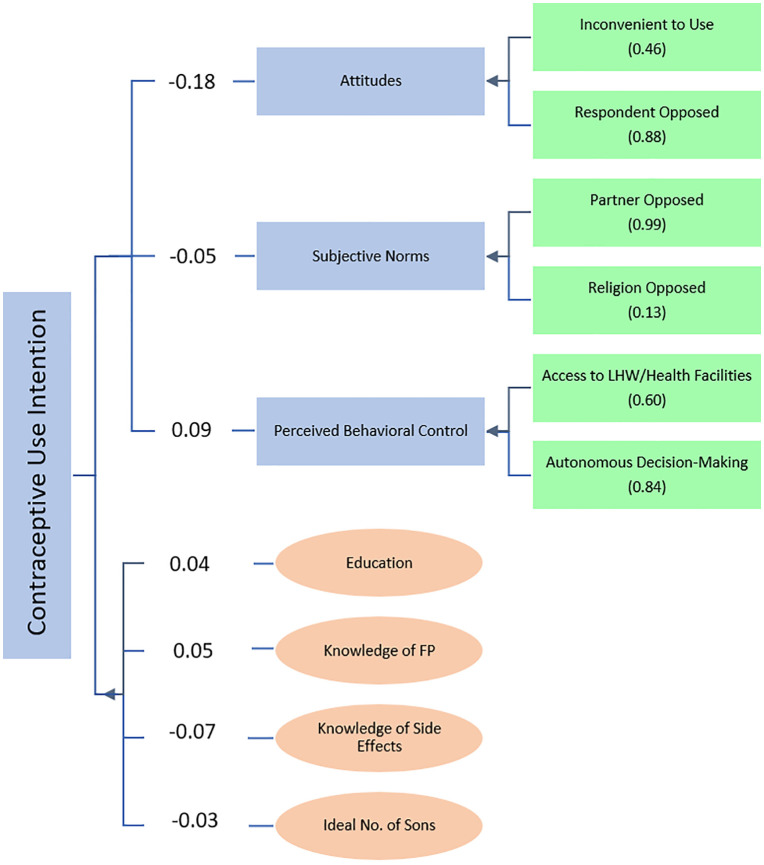
Graphical output of structural equation modeling.

Subjective norms (β = − 0.056, p < 0.001) and perceived behavioral control (β = 0.091, p < 0.001) significantly affect contraceptive use. Subjective norms have a negative relationship with the intention to use contraceptives while Perceived Behavioral Control has a positive relationship with intention to use. The relationship between attitude (β = − 0.175) and intention to contraceptive use was insignificant (Table 5 *and* Fig 3).

Among control variables, wealth, occupation status, and employment after marriage have an insignificant effect on the intention to use contraceptives. Age has a negative and significant (β = − 0.059, p < 0.001), while the woman’s educational attainment has a positive and significant relationship with intention to use (β = 0.040, p < 0.001). The intention to use is negatively and significantly associated with knowledge about side effects (β = −0.069, p < 0.001) and positively and significantly associated with knowledge about family planning (β = 0.049, p < 0.001). Furthermore, the influence of fertility preferences, including births in the last five years, the ideal number of sons, and the total number of children, on contraceptive use intention were evaluated. Births in the last 5 years (β = 0.032, p < 0.001) and the total number of children (β = 0.027, p < 0.001) increase the intention to use.

## 4. Discussion

This study applied the Theory of Planned Behavior (TPB) to identify psychosocial determinants of contraceptive intention among women in Pakistan using nationally representative data. The results provide strong evidence that TPB offers a useful framework for understanding how social pressures, autonomy, and individual beliefs shape reproductive decision-making in a context characterized by patriarchal norms and constrained agency. Notably, subjective norms and perceived behavioral control (PBC) emerged as the strongest predictors of contraceptive intention, while personal attitudes showed no significant association. Together, these findings illuminate how behavioral pathways operate in collectivist societies and highlight critical areas for programmatic intervention.

The negative association between subjective norms and contraceptive intention underscores the powerful role of social expectations in shaping women’s reproductive choices. Even though only a small proportion of husbands or family members explicitly oppose contraceptive use, subtle forms of disapproval—including lack of support, passive resistance, and gendered expectations—may be sufficient to deter women from intending to use contraception [19]. This aligns with evidence from Burkina Faso, Nigeria, and Pakistan showing that women often defer to their husband’s preferences or extended family expectations, even when their own attitudes are favorable [20–22]. In such environments, interspousal communication and gender power dynamics become central determinants of reproductive autonomy. The findings therefore reaffirm TPB’s premise that subjective norms can substantially shape intention, while illustrating the heightened sensitivity of women’s contraceptive decisions to social influence in patriarchal settings.

Perceived behavioral control was also a significant predictor of contraceptive intention, highlighting the importance of autonomy and access to services. Women who had greater decision-making power or access to health facilities and Lady Health Workers (LHWs) were more confident in their ability to use contraception and therefore more likely to intend to use it. This finding is consistent with prior research from Pakistan showing that women often require permission from husbands, elders, or in-laws to seek care, and that limited mobility or lack of nearby FP services reduces their likelihood of adopting contraception [23–28]. In this study, more than half of women reported autonomy in FP decision-making, and a large proportion had access to nearby services, which may partly explain the positive influence of PBC. These results validate TPB’s assertion that perceived control over one’s environment enhances behavioral intention, and they also indicate that improving service access and decision-making autonomy could meaningfully strengthen contraceptive uptake.

In contrast, attitudes toward contraception did not significantly predict intention—an unexpected finding given TPB’s theoretical assumptions and evidence from other LMICs. However, this result is highly plausible within Pakistan’s sociocultural context. In societies where women’s personal preferences are often subordinated to family expectations, individual attitudes may have limited behavioral relevance. Even women with favorable views of contraception may refrain from intending to use it if they anticipate disapproval from their husbands or if they lack the autonomy to access services. Similar patterns have been reported in studies from Uganda and Nigeria, where positive attitudes toward contraception did not translate into intention because of familial pressure, misinformation, or religious norms [29]. This divergence between attitudes and intention reinforces the argument that TPB constructs may operate differently in collectivist societies and that subjective norms and perceived control may outweigh personal beliefs in shaping reproductive choices.

Socioeconomic factors further contributed to variations in contraceptive intention. Education and knowledge of FP emerged as enabling factors, reflecting the role of awareness, empowerment, and exposure to information in shaping intention. Conversely, greater knowledge of side effects was associated with reduced intention, underscoring the impact of fear, misinformation, and negative past experiences—patterns well documented in FP literature [30]. Fertility preferences, including number of births and total children, also raised intention, suggesting that women may consider contraception more seriously only after achieving desired family size. Age showed a negative association, likely reflecting declining fertility desires or perceptions that contraceptive use is unnecessary in later reproductive years [31,32].

Taken together, these findings illustrate how TPB constructs interact with sociocultural structures to shape reproductive decision-making. In settings like Pakistan, subjective norms and perceived behavioral control appear to have greater predictive power than individual attitudes—highlighting the importance of targeting social influence, gender power relations, and structural barriers when designing FP interventions. The study also demonstrates that TPB remains a valuable framework in LMIC contexts but requires contextual interpretation, particularly where individual agency is constrained by collective norms.

Overall, the study contributes to the literature by empirically testing TPB using a nationally representative dataset and demonstrating both its applicability and its limitations in a highly contextualized environment. Strengthening women’s autonomy, improving access to FP services, engaging men and family members, and addressing misinformation emerge as key pathways for enhancing contraceptive intentions and subsequent use. These insights provide an evidence-based foundation for designing culturally grounded, behaviourally informed FP programs in Pakistan and similar settings.

### 4.1. Conclusion

This study applied the Theory of Planned Behavior (TPB) to examine psychosocial determinants of contraceptive intention among women in Pakistan. Using nationally representative data, the analysis demonstrates that subjective norms and perceived behavioral control—rather than personal attitudes—are the strongest predictors of contraceptive intention. These findings highlight that reproductive decision-making in Pakistan is shaped more by social expectations, gender power dynamics, and structural constraints than by individual evaluations of contraceptive use. In a context where women’s autonomy is limited and decision-making is embedded within patriarchal family structures, intention is strongly influenced by the attitudes of husbands, in-laws, and community norms, as well as by women’s perceived ability to access FP services.

By empirically validating TPB in this setting, the study contributes to a deeper understanding of how behavioral constructs operate within collectivist societies. The results underscore the importance of moving beyond individual-level interventions and integrating social, relational, and structural considerations into family planning programming. Thus, TPB offers a valuable framework for designing culturally grounded interventions that address both psychosocial barriers and contextual realities faced by Pakistani women.

### 4.2. Policy Recommendations

Based on the study findings, several policy and programmatic priorities emerge:

1. **Strengthen male and family engagement in FP counselling**

Subjective norms strongly influence intention. Programs should involve husbands, mothers-in-law, and influential community members to create supportive social environments for women’s contraceptive decisions.

1. **Improve women’s autonomy and decision-making power**

Interventions that promote joint decision-making, build negotiation skills, and support women’s agency—particularly within marital and extended family structures—can enhance perceived control.

1. **Expand access to culturally acceptable FP services**

Doorstep delivery through Lady Health Workers, community-based outreach, and youth-friendly services can significantly improve perceived behavioral control.

1. **Address misinformation and fear of side effects**

Evidence-based FP counseling, mass media campaigns, and targeted myth-busting interventions are essential to counter misconceptions that reduce intention.

1. **Tailor FP interventions to life stage and fertility preferences**

Women’s intention to use contraception increases after achieving desired family size. Programs should target newly married women, postpartum women, and women with unmet FP needs through segmented strategies.

### 4.3. Study limitations

This study has important limitations that must be acknowledged. First, the analysis is based on the Pakistan Demographic and Health Survey (PDHS) 2017–18, which—although still the most recent nationally representative dataset—limits inclusion of newer FP trends. Second, TPB constructs were operationalized using available DHS indicators, which are binary and may not capture the full complexity of psychosocial factors such as motivation, perceived stigma, or interpersonal communication quality. Third, the cross-sectional nature of the data restricts causal inference; we cannot determine whether intention leads to subsequent contraceptive use. Finally, the reliance on self-reported measures may introduce recall or social desirability bias.

### 4.4. Future directions

Future studies should incorporate primary data collection using multi-item validated TPB scales, qualitative exploration of spousal and family pressures, and longitudinal designs to examine the intention–behavior gap. Mixed-methods research could provide deeper insights into how norms, autonomy, and service access interact to shape contraceptive behaviors across diverse communities. Additionally, testing extended models—such as integrating perceived stigma, interspousal communication quality, or cultural norms—may strengthen predictive power and enhance contextual relevance.

## Supporting information

S1 File Stata latest complete model file 2. (DTA)
